# Decontamination and Annotation of the Draft Genome Sequence of the Oomycete Lagenidium giganteum ARSEF 373

**DOI:** 10.1128/mra.01346-22

**Published:** 2023-04-13

**Authors:** William R. Morgan, Aurélien Tartar

**Affiliations:** a Department of Biology, The College of Wooster, Wooster, Ohio, USA; b Department of Biological Sciences, Nova Southeastern University, Fort Lauderdale, Florida, USA; Indiana University, Bloomington

## Abstract

Scaffolds of a previously published Lagenidium giganteum ARSEF 373 genome assembly found at GenBank were filtered to remove contaminating sequences. Genome annotation of the 437 scaffolds (total length, 56.2 MB; GC content, 58.8%) with confirmed *L. giganteum* sequences identified 13,069 potential protein-coding genes, encoding at least 737 predicted secreted proteins and >100 putative translocated effectors.

## ANNOUNCEMENT

The oomycete Lagenidium giganteum is recognized as a biocontrol agent for mosquitoes, especially Aedes aegypti (1). With a goal of developing environmentally sustainable compounds for controlling mosquito populations, genomic studies have been undertaken to better understand the molecular mechanism by which *L. giganteum* infects mosquito larvae (2, 3).

An unannotated *L. giganteum* ARSEF 373 whole-genome assembly (accession number GCA_002286825.1) is available in the NCBI Genome database (4), but we found that both *cox1* gene sequences in this assembly differ extensively (~92% identity) from *cox1* sequences previously amplified from *L. giganteum* isolates (5). Furthermore, one was nearly identical (>99.9%) to a DNA sequence (SPLM01000079.1) in the oomycete Pythium oligandrum, a close relative of *Lagenidium* (6), suggesting the presence of contaminating DNA sequences.

To explore the extent of the contamination, we aligned the scaffold sequences (GenBank: NSDO00000000.1) using NCBI BLASTN v2.11.0 with default parameters to collections of *Pythium oligandrum* (NCBI txid41045) and *L. giganteum* (txid4803) DNA and mRNA sequences. Of the 2,779 scaffolds, 689 extensively matched *P. oligandrum* sequences (>99% identity, >1,000 bp), indicating extensive contamination. Of interest here, 437 scaffolds (56.2 Mb; GC content, 54.84%; *N*_50_, 202 kb) extensively match *L. giganteum* sequences.

The 437 scaffolds with confirmed *L. giganteum* sequences were annotated using Maker2 v3.01.03 (7, 8). The annotation evidence included 34,733 *L. giganteum* PacBio transcriptome sequencing (RNA-seq) reads (mean length, 1,122.6 bp), 608,747 *Oomycota* (NCBI txid4762) mRNA sequences retrieved from NCBI’s nucleotide database (accessed June 2021; mean length, 830.8 bp), and 83 *L. giganteum* (txid4803) and 1,303,914 *Oomycota* (txid4762) protein sequences retrieved from NCBI’s Identical Protein Groups database (accessed June 2021; mean lengths, 268.8 amino acids [aa] and 423.1aa, respectively).

After repeat sequences were masked using an *L. giganteum*-specific repeat library built with RepeatModeler v2.0.2a (9), protein-coding gene predictions were inferred in the first round of Maker using mRNA alignments and protein homology (8). After training Augustus v3.3.3 (10) and SNAP v2019-06-03 (11) with the first-round gene predictions, the optimized parameters were used in MAKER for a round of *ab initio* gene prediction. Finally, putative gene functions were assigned based on protein homology to UniProt/Swiss-Prot entries (12). After quality filtering, the final build contained 13,069 annotated genes.

Several measures indicate a good quality genome annotation. First, the genome size and number of predicted genes is comparable to those of related genomes (13, 14). Second, 96% of gene annotations had a low (<0.5) annotation edit distance (15), and 54% had a recognizable protein domain, exceeding MAKER’s “rule-of-thumb” values (90% and 50%, respectively) for a well-annotated genome (8). Furthermore, analysis using BUSCO v5.2.2 (16) found that the *L. giganteum* protein set contained complete copies of 92% of 100 stramenopile and 57.4% of 758 fungal “core” orthologs, comparable to genome annotation projects in related species (17). Comparable to *Pythium* genomes, the *L. giganteum* genome include 737 genes predicted to encode secreted proteins (18), with over 100 similar to RXLR or Crinkler pathogen effectors (19–22). This annotated genome will enhance future studies investigating the mechanisms of *L. giganteum* pathogenesis and support the development of more sustainable approaches for controlling mosquito populations.

### Data availability.

This whole-genome shotgun project has been deposited at DDBJ/ENA/GenBank under the accession number DAKRPA000000000 and the BioProject accession number PRJNA256125. The version described in this paper is DAKRPA010000000. The raw reads were deposited in the SRA under accession number SRR21186961. The repeat library and other supporting files are available at https://doi.org/10.6084/m9.figshare.c.6466708.
